# Electric-field control of magnetic moment in Pd

**DOI:** 10.1038/srep14303

**Published:** 2015-09-22

**Authors:** Aya Obinata, Yuki Hibino, Daichi Hayakawa, Tomohiro Koyama, Kazumoto Miwa, Shimpei Ono, Daichi Chiba

**Affiliations:** 1 Department of Applied Physics, Faculty of Engineering, The University of Tokyo, Bunkyo, Tokyo 113-8656, Japan; 2 Central Research Institute of Electric Power Industry, Komae, Tokyo 201-8511, Japan

## Abstract

Several magnetic properties have recently become tunable with an applied electric field. Particularly, electrically controlled magnetic phase transitions and/or magnetic moments have attracted attention because they are the most fundamental parameters in ferromagnetic materials. In this study, we showed that an electric field can be used to control the magnetic moment in films made of Pd, usually a non-magnetic element. Pd ultra-thin films were deposited on ferromagnetic Pt/Co layers. In the Pd layer, a ferromagnetically ordered magnetic moment was induced by the ferromagnetic proximity effect. By applying an electric field to the ferromagnetic surface of this Pd layer, a clear change was observed in the magnetic moment, which was measured directly using a superconducting quantum interference device magnetometer. The results indicate that magnetic moments extrinsically induced in non-magnetic elements by the proximity effect, as well as an intrinsically induced magnetic moments in ferromagnetic elements, as reported previously, are electrically tunable. The results of this study suggest a new avenue for answering the fundamental question of “can an electric field make naturally non-magnetic materials ferromagnetic?”

Application of an electric fields is a useful tool for controlling the magnetism of magnetic materials^1^,^2^,^3^,^4^,^5^,^6^,^7^,^8^,^9^,^10^,^11^,^12^,^13^,^14^,^15^,^16^,^17^,^18^,^19^,^20^,^21^,^22^,^23^,^24^,^25^,^26^,^27^,^28^ as well as the conductivity of semiconductors. Many studies have been reported, including that of ferromagnetic metals at room temperature^4^,^7^,^14^,^15^,^17^,^18^,^19^,^20^,^21^,^22^,^23^,^24^,^25^,^26^,^27^, using a capacitor structure, with which carrier density can be controlled by applying a gate voltage. Electric-field-induced magnetization switching may drastically reduce power consumption in magnetic recording devices^2^,^7^,^8^,^13^,^22^,^23^,^24^,^25^,^26^,^27^.

The changes in material’s magnetic properties induced by an electric field are considered to be related to a change in carrier density and, in turn, a shift of the Fermi level. Therefore, we expect that the application of an electric field may make naturally non-magnetic materials ferromagnetic^5^,^12^. Pt and Pd are usually non-magnetic metals that nearly satisfy the Stoner criterion^29^,^30^,^31^. *Ab initio* calculations have shown that the peak of the density of the states of bulk non-magnetic Pt or Pd is located at an energy near the Fermi level^29^, suggesting that an applied electric field may affect the magnetic state in these materials^3^,^5^,^12^,^26^. It is also known that a magnetic moment is induced by the ferromagnetic proximity effect in a Pt or Pd layer deposited on a ferromagnetic metal layer^32^,^33^,^34^,^35^,^36^,^37^. In the present study, we investigated whether the magnetic moment induced in Pd can be electrically controlled. We prepared Pt/Co/Pd structures and observed a clear change in their magnetic moments as a result of applying an electric field to the ferromagnetic surface of the Pd layer. The results indicate that the magnetic moment extrinsically induced in non-magnetic elements by the proximity effect is electrically tunable.

## Results

### Ferromagnetic proximity effect on Pd deposited on Pt/Co layers

**Figure 1:**
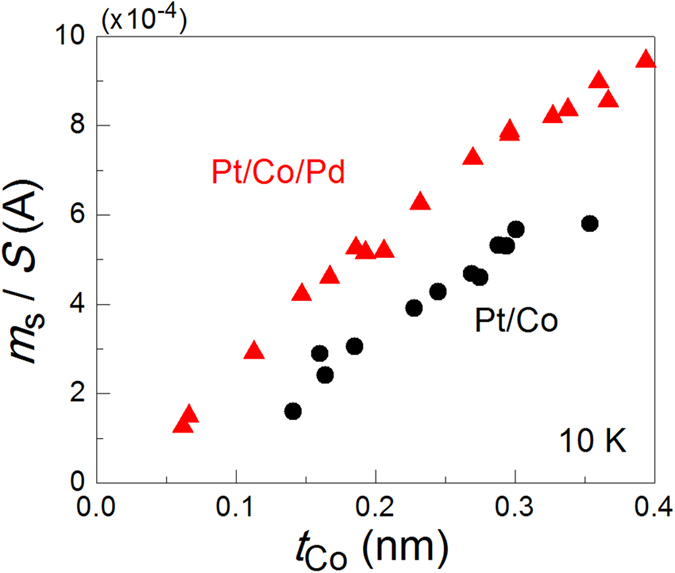
Co thickness *t*_Co_ dependence of the perpendicular component of the saturation magnetic moment per unit area at 10 K (*m*_s_/*S*) for Pt/Co (black circles) and Pt/Co/Pd (red triangles) samples. The *m*_s_/*S* values of the Pt/Co/Pd samples were greater than those of the Pt/Co samples for all *t*_Co_ values, indicating that a magnetic moment was induced in the Pd layer because of the ferromagnetic proximity effect.

Ta(~3 nm)/Pt(4.1 nm)/Co(*t*_Co_)/Pd(1.7 nm)/MgO(~2 nm) layers (Pt/Co/Pd samples) from the substrate side were deposited on an intrinsic Si substrate using rf sputtering. Samples without a Pd layer (Pt/Co samples) were also prepared as reference samples to confirm that the magnetic moment was induced in a Pd layer of Pt/Co/Pd samples by the ferromagnetic proximity effect before applying an electric field. Pd and Pt layers were confirmed to have an fcc (111) texture using X-ray diffraction measurement. All of the Pt/Co/Pd and Pt/Co samples we used in this study were confirmed to have perpendicular magnetic anisotropy. Figure 1 shows the *t*_Co_ dependence of the saturation magnetic moment per unit area (*m*_s_/*S*) for the both samples. As shown in the figure, the *m*_s_/*S* values of the Pt/Co/Pd samples were greater than those of the Pt/Co samples for all *t*_Co_ values, indicating that a magnetic moment was induced in the Pd layer. Assuming that the magnetic moment is uniformly induced in the entire Pd layer, the induced magnetic moment per Pd atom is calculated to be ~0.1*μ*_B_ (*μ*_B_ is the Bohr magneton), the order of which is in good agreement with previous studies^32^,^34^,^35^. The Pd layer thickness dependence indicates that the magnetic moment induced in the Pd layer saturates at *t*_Pd_ ~ 2 nm (not shown), suggesting that ~2 nm is the distance limit of the proximity effect from the Co/Pd interface^35^. Thus, in the present sample (*t*_Pd_ = 1.7 nm < 2 nm), a finite magnetic moment is expected to be induced in the uppermost Pd atomic layer, but the magnetic moment per Pd atom there is considered to be less than 0.1*μ*_B_ because the magnetic moment induced by the ferromagnetic proximity effect is known to decrease with increasing distance from the interface^34^,^36^.

### Fabrication of the devices and experimental setup for magnetic moment measurement under applied electric field

**Figure 2:**
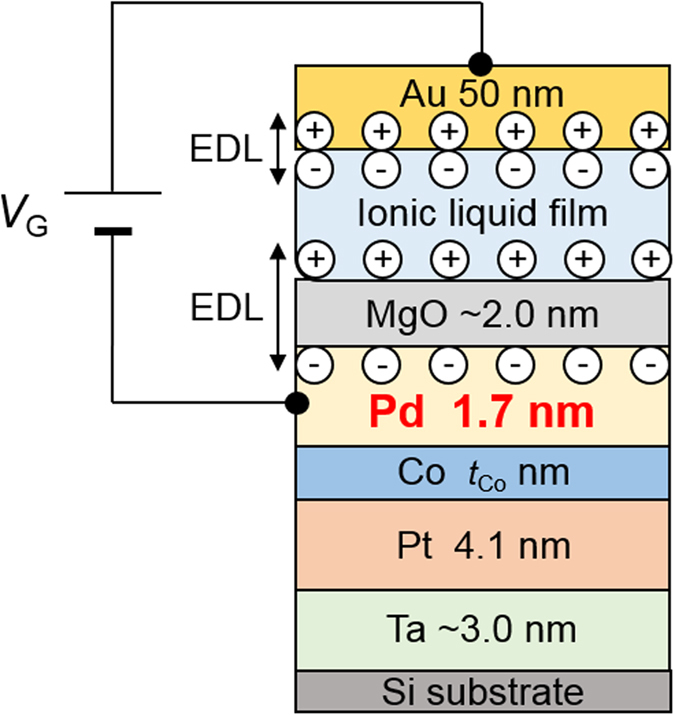
Schematic cross section of the device. A polymer film containing an ionic liquid (ionic liquid film) was used to apply an electric field to the surface of the Pd layer, in which a magnetic moment was induced. By applying a gate voltage *V*_G_, a pair of electric double layers (EDL) was formed at the top and bottom interfaces of the ionic liquid film. The formation of the bottom EDL resulted in a change in the electron density at the surface of the Pd layer. Because of Thomas–Fermi screening, only the electron density at the uppermost Pd layer was expected to be modulated.

An electric-double-layer (EDL) capacitor structure was used to modulate the electron density in the Pd surface^3^,^4^,^11^,^15^,^16^,^18^,^26^. The structure consisted of an Au gate electrode, a polymer film containing an ionic liquid (ionic liquid film)^18^, and a Pt/Co/Pd sample (Fig. 2). Two Pt/Co/Pd samples with *t*_Co_ values of 0.10 and 0.19 nm (samples A and B, respectively) were used in the experiment. The ionic liquid we used was composed of an N,N,N-trimethyl-N-propylammonium (TMPA^+^) cation and a bis(trifluoromethylsulfonyl)imide (TFSI^-^) anion. The EDL capacitor was fabricated by simply placing the ionic liquid film with the evaporated Au gate electrode (50 nm) on the Pt/Co/Pd sample. The area covered by the ionic liquid film (*S*_ion-film_) was slightly less than the total area of the Pt/Co/Pd sample (*S*_total_). Au wires were connected to the Au gate electrode and the Pt/Co/Pd metallic layers to apply a gate voltage *V*_G_ between them. A positive *V*_G_ corresponded to the direction of increase in the electron density at the Pd surface. The device was introduced into a superconducting quantum interference device magnetometer to measure the magnetic moment directly under the application of *V*_G_^6^,^18^.

### Magnetic moment under electric field

**Figure 3:**
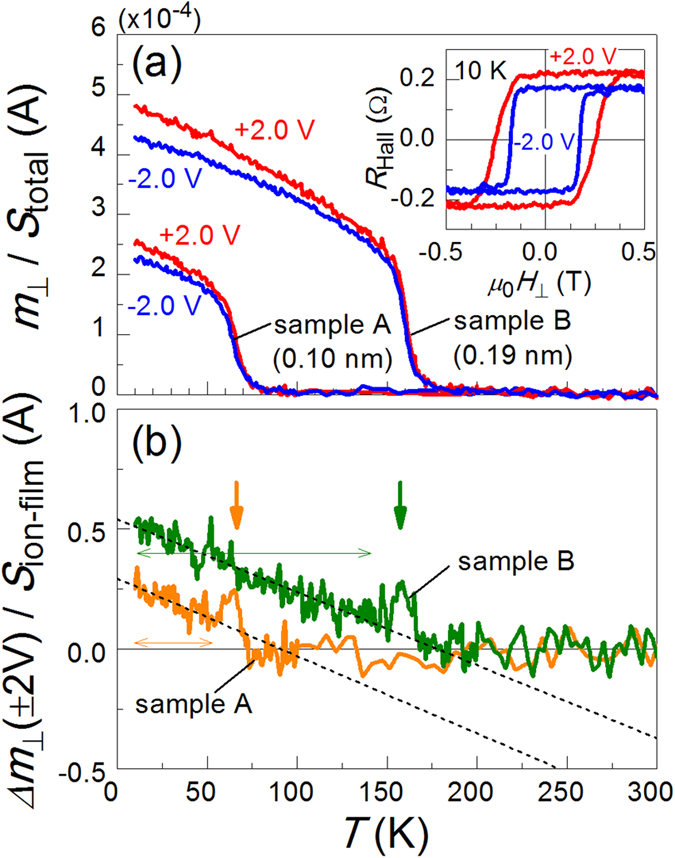
(**a**) Temperature *T* dependence of the perpendicular component of the magnetic moment *m*_┴_ normalised by the total sample area *S*_total_ at *V*_G_ = ± 2.0 V for samples A (Co layer thickness *t*_Co_ = 0.10 nm) and B (*t*_Co_ = 0.19 nm). Up to 100 K (200 K) for sample A (B), the temperature was increased by 2 K/min, and the averaged *m*_┴_ were measured every 1 K. For averaging, two data points were acquired in the range of 0.2 K, which was much smaller than the temperature interval (1 K) of each averaged *m*_┴_. The inset shows the hysteresis loops observed in the Hall resistance (*R*_Hall_) at 10 K at *V*_G_ = ± 2.0 V for sample A, from which the squareness ratio of the loops was confirmed to be ~1, regardless of the value of *V*_G_. (**b**) The difference between *m*_┴_ for *V*_G_ = + 2.0 and –2.0 V *Δm*_┴_(±2 V) is shown as a function of temperature for samples A and B. The *Δm*_┴_ (±2 V) shown on the vertical axis was divided by the area covered by the ionic liquid film (*S*_ion-film_). The dashed lines indicate the results of the linear fitting using the data within the range indicated by the double-headed horizontal arrows.

Figure 3(a) shows the temperature *T* dependence of the perpendicular component of the magnetic moment *m*_┴_ divided by *S*_total_ for both samples A and B under *V*_G_ = + 2.0 and −2.0 V. After *V*_G_ was changed at 300 K under a perpendicular magnetic field *μ*_0_*H*_┴_ of ~20 mT, *T* was decreased to 10 K. Subsequently, *μ*_0_*H*_┴_ was reduced to nearly zero (1.5 ± 0.1 mT), and the *m*_┴_ shown in Fig. 3(a) was measured by increasing *T*. Below the Curie temperature *T*_C_, a clear difference in *m*_┴_/*S*_total_ was observed with the change in *V*_G_ for both samples: a positive (negative) *V*_G_, *i.e.*, larger (smaller) electron density at the Pd surface, resulted in a larger (smaller) *m*_┴_/*S*_total_. The difference in *m*_┴_ between *V*_G_ = + 2.0 and –2.0 V (*Δm*_┴_( ± 2 V)) was found to increase linearly with decreasing temperature, as shown in Fig. 3(b), in which *Δm*_┴_(±2 V) was divided by *S*_ion-film_ because the magnetic moment should be modulated in the area covered by the ionic liquid film. It should be noted that *m*_┴_ was nearly equal to *m*_s_ because the squareness ratio *m*_┴_/*m*_s_ of the hysteresis loops was ~1 under *V*_G_ = ± 2.0 V at a temperature of at least 10 K, as indicated in the inset of Fig. 3(a). The anomalous Hall effect was used to detect the hysteresis loop under the application of *V*_G_. Although the change in *T*_C_ up to 100 K was reported for a Pt/Co sample with a similar device structure^18^, *T*_C_ was not clearly dependent on *V*_G_ in the present Pt/Co/Pd samples. *Δm*_┴_( ± 2 V) deviates significantly from the linear fitting immediately before it intercepts the horizontal axis, as indicated by the downward arrows in Fig. 3(b), which might imply the occurrence of a small change in *T*_C_.

## Discussion

**Figure 4:**
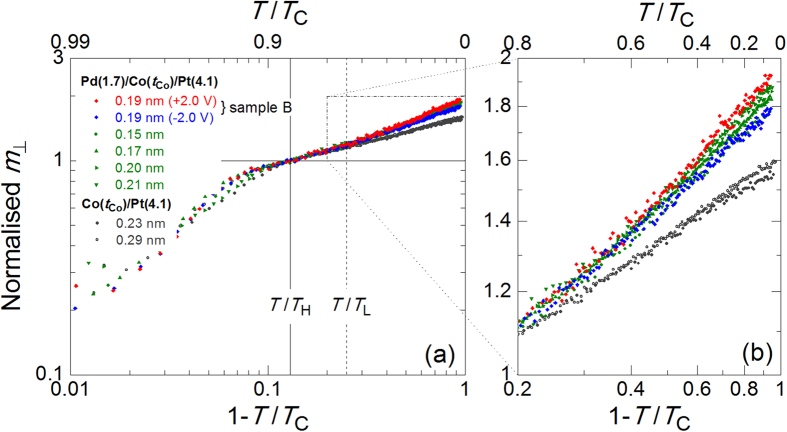
(**a**) Double-logarithmic plot of the normalised *m*_┴_ as a function of 1 − *T*/*T*_C_ for several samples listed in Table 1. (**b**) Magnified view of the region indicated by the dashed-dotted line in (**a**). *T*/*T*_H_ and *T*/*T*_L_ in the figure indicates the range for the fitting performed to obtain the critical exponent *β*. The values of *m*_┴_ on the vertical axis were normalised by the value at *T*/*T*_H_. The magnetic moment in the Pt/Co/Pd samples increased more rapidly than that in the Pt/Co samples as 1 − *T*/*T*_C_ increased (in other words, as the temperature was reduced). The data points for the Pt/Co/Pd samples to which an electric field were not applied (green symbols) were confirmed to lie almost entirely between the ones obtained under the application of positive and negative *V*_G_ to sample B (red and blue symbols) at a temperature *T* < *T*_L_.

We first analysed the temperature dependence of *m*_┴_ for the Pt/Co/Pd and Pt/Co samples. The reduction in the magnetic moment *m* near *T*_C_ can be quantified using a critical exponent *β* as *m* ~ (1 − *T*/*T*_C_)^β^. Figure 4(a) shows a double-logarithmic plot of the normalised *m*_┴_ as a function of 1 − *T*/*T*_C_ for several samples with *T*_C_ in the range of 157–341 K, including sample B, under an applied electric field (see Table 1 for details of the samples). The value of *β* was determined from the slope of the linear fitting to the data^37^,^38^ in the range of *T*_L_/*T*_C_ (=0.75) to *T*_H_/*T*_C_ (=0.87) (see Methods for details of the analysis). The value of *m*_┴_ in the figure was normalised by its value at *T*_H_/*T*. The value of *β* was ~0.2 in the Pt/Co samples and ranged from 0.22 to 0.30 in the Pt/Co/Pd samples. From the magnified figure shown in Fig. 4(b), one can clearly see that as 1 − *T*/*T*_C_ increased (in other words, as the temperature decreased), the difference in the normalised *m*_┴_ values between the Pt/Co/Pd and Pt/Co samples increased, *i.e.*, the magnetic moment in the Pt/Co/Pd samples increased more rapidly at lower temperatures. This behaviour occurred because the *β* value of the Pt/Co/Pd samples was higher than that of the Pt/Co samples and/or because the magnetic moment in the ferromagnetic Pd layer is greater at lower temperatures^35^,^37^. The latter factor is most likely dominant here because the linearity of the normalised *m*_┴_ degrades as 1 − *T*/*T*_C_ increases in the double-logarithmic scale. Other important points to consider in understanding the electric-field effect observed in this study are as follows. (i) A larger change in the magnetic moment was observed with the application of *V*_G_ in the Pt/Co/Pd samples at lower temperatures, as shown in Figs 3(b) and 4(b). (ii) The data points for the Pt/Co/Pd samples without an applied electric field (indicated by the green symbols in Fig. 4) are almost entirely located between the ones obtained under positive and negative *V*_G_ (indicated by the red and blue symbols) at any temperature *T* < *T*_L_. (iii) As shown in Fig. 3(b), the difference between *m*_┴_ under positive and negative *V*_G_ (*Δm*_┴_(±2 V)) increased linearly with decreasing temperature. This can probably be attributed to the linear temperature dependence of the magnetic susceptibility of Pd deposited on the layer consisting of the ferromagnetic element^35^,^37^. These experimental results, which show good reproducibility in similar structures (see Methods), demonstrate that the induced magnetic moment at the surface of the Pd layer was increased and decreased by the application of positive and negative *V*_G_, respectively.

**Figure 5:**
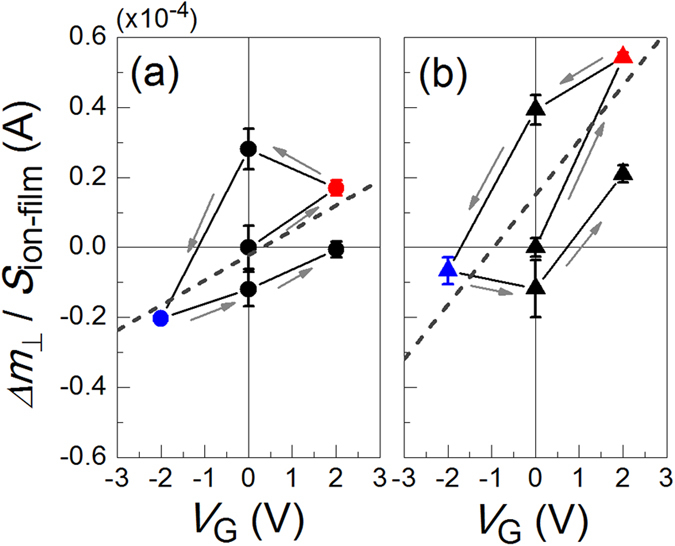
Change in the perpendicular component of the magnetic moment (*Δm*_┴_) at 10 K normalised by *S*_ion-film_ for samples (a) A and (b) B as a function of *V*_G_. *V*_G_ was changed at 300 K, and the temperature was reduced to measure each value of *m*_┴_ at 10 K. The dashed line indicates the linear fitting to the averaged *Δm*_┴_/*S* at each *V*_G_, from which the change in the magnetic moment per Pd atom was determined.

We next analysed the change in *m*_┴_ (*Δm*_┴_) for samples A and B upon application of an electric field. Figures 5(a,b) show *Δm*_┴_/*S*_ion-film_ at 10 K for both samples as a function of *V*_G_. Each data point was obtained from the temperature dependence of *m*_┴_ at 10 K under each *V*_G_. *V*_G_ was applied in the order indicated by the arrows in the figures. Although hysteresis behaviour was observed with round-trip *V*_G_ application, the tendency of *Δm*_┴_ to increase (decrease) with positive (negative) *V*_G_ application was reproduced in both samples. The change in the magnetic moment per Pd atom was determined from the linear fitting, as shown in the figures, based on the following assumptions. Pd has an fcc (111) structure, and only the magnetic moment in the uppermost atomic layer was changed because only the electron density there was changed attributed to Thomas–Fermi screening (see Methods for details of the capacitance measurement). The calculated value of the magnetic moment for sample A (B) was 0.050 ± 0.023*μ*_B_ (0.078 ± 0.013*μ*_B_) per *V*_G_ of 1.0 V. The change in the electron number *ΔN* for sample A (B) per Pd atom and per *V*_G_ of 1.0 V was calculated to be 0.049 (0.034). *ΔN* was determined from the capacitance *C* of the devices divided by *S*_ion-film_.

According to the *ab initio* calculation for Pd, the density-of-states peak appears at an energy level slightly lower than the Fermi level^29^. Thus, a decrease in the electron density should theoretically lead to an increase in the magnetic moment^5^,^12^. Our results, however, were the opposite. Support from theoretical calculations is clearly needed to understand our results. The state of the Pd in our sample structure was different from that in the bulk case in the following respects: (1) the magnetic moment was already induced in the Pd layer (thus, spin splitting was already induced); and (2) the Pd layer was very thin, and interfaces were formed between the Co and MgO layers. In addition, we note that in a Pt/Co system, in which the electric field induced changes in *T*_C_ and a magnetic moment was reported^17^,^18^, *ab initio* calculations indicated that the number of 3*d* electrons can be decreased even when an electric field is applied in the direction of increase in the total electron number because the number of *sp* electrons increases^28^. This explains the discrepancy between the experimental results and the outcome suggested by the Slater–Pauling curve for the Co case. This scenario may be applicable even to the present 4*d* electron system (Pd). Furthermore, reversible chemical effects, such as the migration of oxygen atoms from the MgO^27^ cap or of hydrogen absorption in the Pd layer^39^ by *V*_G_ applications might be related to a change in the electron state and thus to a change in the magnetic moment induced in Pd. From an experimental perspective, the next challenge is to induce ferromagnetism electrically in naturally non-magnetic materials beyond that attributable to the ferromagnetic proximity effect.

## Methods

### Determination of *T*_C_

**Figure 6:**
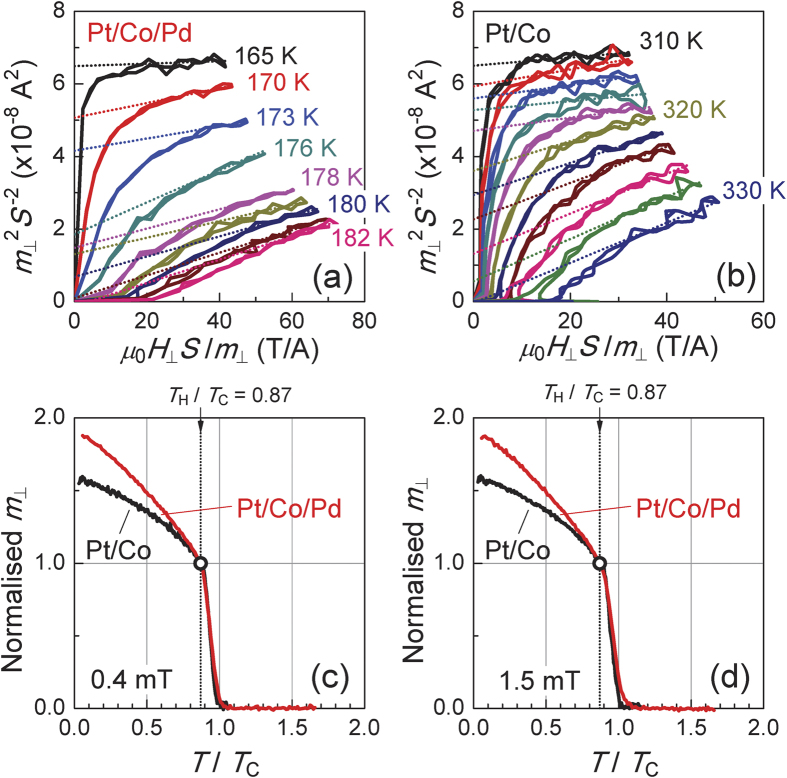
(**a**,**b**) The Arrott plots for the Pt/Co/Pd and the Pt/Co reference samples, respectively. (**c**,**d**) The normalised temperature (*T*/*T*_C_) dependence of the normalised magnetic moment (*m*_┴_) for both reference samples at 0.4 (±0.1) mT and 1.5 (±0.1) mT, respectively. The vertical axes in (**c**,**d**) were normalised by the *m*_┴_ value at *T*/*T*_C_ = 0.87.

**Figure 7:**
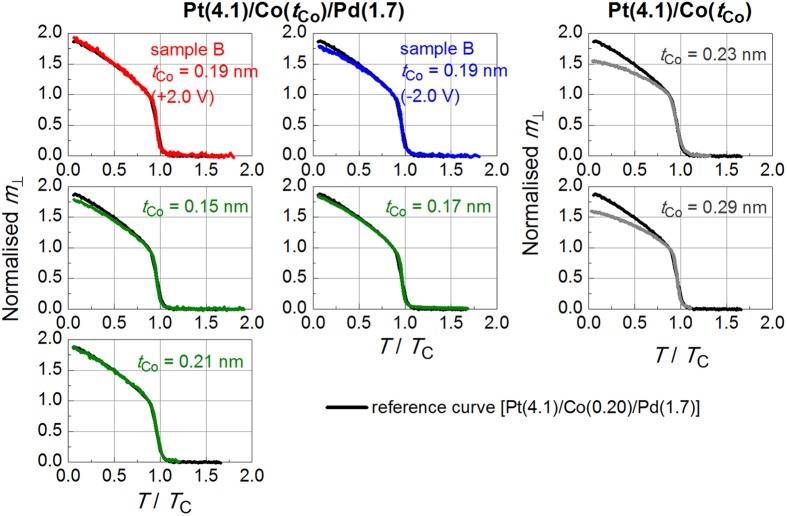
*T*/*T*_C_ dependence of the normalised *m*_┴_ for all samples listed in Table 1. The black line indicates the results obtained for the Pt/Co/Pd reference sample. The results are indicated by coloured lines. All *m*_┴_ was measured at 1.5 (±0.1) mT and the vertical axes were normalised by the *m*_┴_ value at *T*/*T*_C_ = 0.87.

Figures 6(a,b) show the Arrott plots^40^ for two reference samples, a Pt/Co/Pd sample with *t*_Co_ = 0.20 nm and *t*_Pd_ = 1.7 nm and a Pt/Co sample with *t*_Co_ = 0.32 nm, respectively. From the plots, the *T*_C_ of the Pt/Co/Pd (the Pt/Co) reference sample was determined to be 181 (329) K. The *T*/*T*_C_ dependence of *m*_┴_ at *μ*_0_*H*_┴_ = 0.4 (±0.1) mT for both reference samples is shown in Fig. 6(c), in which the vertical axis is normalised by an *m*_┴_ value at *T*/*T*_C_ = 0.87. In both samples, *m*_┴_ decreased rapidly below *T*_C_. This was most likely because of the formation of a multi-domain state, as observed in similar Pt/Co samples^17^,^41^. The important point is that, as long as *μ*_0_ *H*_┴_ was the same (see Figs 6(c,d)), the two curves for Pt/Co/Pd and Pt/Co reference samples overlap very well in this temperature region, even though these samples have different sample structures and *T*_C_ values. This suggests that as long as *T*_C_ is known for one reference sample, one can determine the *T*_C_ of another sample by comparing the *m*_┴_–*T* curves. Figure 7 presents a summary of the comparison of the results for the samples listed in Table 1 (coloured line) and the Pt/Co/Pd reference sample (black line). Adjusting the *T*_C_ values of the samples resulted in the normalised curves overlapping well with the reference curve. The *T*_C_ values of the samples listed in Table 1 were determined in this way. The difference in *T*_C_ for these samples determined using the Pt/Co/Pd and Pt/Co reference samples was at most 1–2%. The Arrott–Noakes (AN) plot^42^ may provide more accurate *T*_C_ for the present two-dimensional system. However, the difference in *T*_C_ determined from the Arrott and AN plots is only 1–2%^17^. In comparing many samples without difficulty and confirming the most important finding of this study, *i.e.*, the induced magnetic moment in the Pd layer being increased or decreased by applying an electric field, we believe that the Arrott plot and the above-mentioned way of determining *T*_C_ were useful method. We note that using a similar Pt/Co/Pd sample with a *T*_C_ of ~190 K (not shown in Table 1) confirms the reproducibility of the results, *i.e.*, the data points for the sample without an applied electric field are almost entirely located between the points obtained for similar samples under positive and negative *V*_G_ at any temperature *T* < *T*_L_.

### Determination of the critical exponent *β*

The value of *β* was determined from the slope of the linear line fitted to the normalised *m*_┴_ data in the range between *T*_L_/*T*_C_ (=0.75) and *T*_H_/*T*_C_ (=0.87) on a double-logarithmic scale (see Fig. 4a). The fitting range was determined from the linearity of the data. It was difficult to perform a reliable fitting to the data for sample A because there were not enough data points in the fitting range.

### Capacitance measurement

The capacitance *C* of samples A and B was measured using a capacitance meter and applying an ac voltage with an amplitude of 0.1 V and frequency *f* of 100 Hz. In addition, the *f* dependence of *C* for a similar device showed that *C* increases slightly with decreasing *f*. Although further experiments are needed to determine an accurate value for *ΔN* under a dc gate voltage, the value of *C* estimated using the above experimental results for *f* = 0.01 Hz, which was the lowest value of *f* in the experiment, was used to calculate the *ΔN* described in this report. The values of *C*/*S*_ion-film_ at *f* = 0.01 Hz for samples A and B were 12 and 8.3 μF/cm^2^, respectively.

## Additional Information

**How to cite this article**: Obinata, A. *et al.* Electric-field control of magnetic moment in Pd. *Sci. Rep.* **5**, 14303; doi: 10.1038/srep14303 (2015).

## Figures and Tables

**Table 1:**
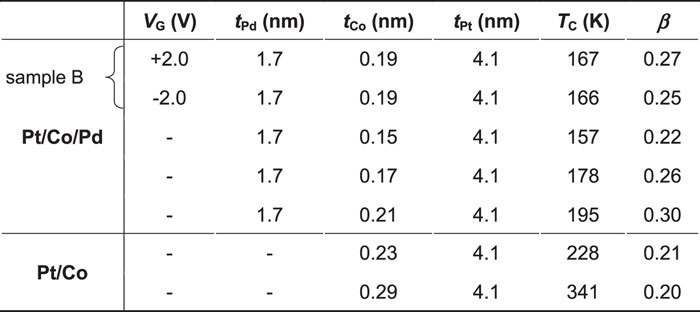
Properties of the Pt/Co/Pd and Pt/Co samples used in Fig. 4.

The thicknesses of the Pd, Co, and Pt layers (*t*_Pd_, *t*_Co_, and *t*_Pt_); *T*_C_; and the critical exponent *β* determined from the fitting (see Methods for details of the determination) are summarised.
