# Erratum: Electric-field control of magnetic moment in Pd

**DOI:** 10.1038/srep15594

**Published:** 2015-10-29

**Authors:** Aya Obinata, Yuki Hibino, Daichi Hayakawa, Tomohiro Koyama, Kazumoto Miwa, Shimpei Ono, Daichi Chiba

Scientific Reports 5: Article number: 14303; 10.1038/srep14303published online: 09222015; updated: 10292015

The original version of this Article contained a typographical error in the received date ‘03 March 2015’ which was incorrectly given as ‘03 March 2014’.

This error has now been corrected in both the PDF and HTML versions of the Article.

